# Multiple Brain Microabscesses and a Lung Abscess Caused by *Streptococcus intermedius* Following COVID-19: A Case Report and Literature Review

**DOI:** 10.3390/idr18040064

**Published:** 2026-06-24

**Authors:** Ryoma Takeda, Kazunori Yamada, Takenori Abe, Tomoyuki Ishigo, Hirohiko Nakamura

**Affiliations:** 1 Department of Pharmacy, Nakamura Memorial Hospital, South 1, West 14, Chuo-ku, Sapporo 060-8570, Hokkaido, Japan; 2 Department of Neurology, Nakamura Memorial Hospital, South 1, West 14, Chuo-ku, Sapporo 060-8570, Hokkaido, Japan; 3 Department of Pharmacy, Sapporo Medical University Hospital, South 1, West 16, Chuo-ku, Sapporo 060-8543, Hokkaido, Japan; 4 Department of Neurosurgery, Nakamura Memorial Hospital, South 1, West 14, Chuo-ku, Sapporo 060-8570, Hokkaido, Japan

**Keywords:** COVID-19, brain abscess, lung abscess, secondary infection, *Streptococcus intermedius*

## Abstract

Background: Secondary bacterial infections are increasingly recognized after coronavirus disease 2019 (COVID-19); however, bacterial abscess formation remains uncommon, and the simultaneous occurrence of brain and lung abscesses has not been previously reported. We report a rare case of *Streptococcus intermedius* infection presenting with multiple brain microabscesses and a lung abscess following COVID-19. Case Presentation: A 75-year-old man with no significant medical history except cholelithiasis experienced persistent fever following a diagnosis of COVID-19 and subsequently developed impaired consciousness 17 days later. Because bacterial meningitis was suspected, he was admitted to a neurology-specialized hospital on the same day. Brain MRI revealed more than 80 small enhancing lesions scattered throughout the brain parenchyma, consistent with multiple microabscesses. Chest CT demonstrated a mass-like lesion in the left lower lobe. Although cerebrospinal fluid cultures were negative, blood cultures obtained on admission yielded *S. intermedius*. Further investigation of the source of infection revealed moderate periodontitis, suggesting the oral cavity as the probable portal of entry. The patient was treated with intravenous antibiotics for eight weeks based on antimicrobial susceptibility testing, resulting in near-complete resolution of the lesions. Conclusions: Although a causal relationship between COVID-19 and abscess formation cannot be established, COVID-19-associated immune and mucosal barrier dysfunction may have contributed to the progression and dissemination of infection in this patient. Clinicians should be aware of the possibility of severe bacterial superinfection when fever or respiratory symptoms related to COVID-19 persist, even in patients without overt immunocompromise, particularly in those with pre-existing oral infections.

## 1. Introduction

Secondary bacterial infections are increasingly recognized after coronavirus disease 2019 (COVID-19), and several studies have demonstrated an elevated risk of invasive bacterial disease [1]. Although several case reports have described either lung or brain abscesses following COVID-19 [2,3], the simultaneous development of both types of abscesses has not been documented. *Streptococcus intermedius* is a well-known pathogen that frequently causes brain abscesses; however, such abscesses are typically solitary rather than markedly multiple [4]. Reports describing extensive multiple microabscesses, such as the more than 80 lesions observed in the present case, are exceedingly rare. Here, we report a case of *S. intermedius*-associated multiple brain microabscesses and a lung abscess that developed 17 days after a diagnosis of COVID-19.

## 2. Case Presentation

A 75-year-old man with a medical history notable only for cholelithiasis had otherwise been in good health. He had no smoking or drinking habits. Twenty days before admission, he developed low-grade fever, cough, and sore throat, and was diagnosed with COVID-19 at a local clinic. No antiviral medication was prescribed. His symptoms persisted during home recovery. Seventeen days after the COVID-19 diagnosis, he developed impaired consciousness and a high fever of 39 °C. He was transferred to another hospital by ambulance, where a lumbar puncture suggested meningitis, and was subsequently admitted to our neurology-specialized hospital on the same day. He had received vaccinations for COVID-19 several times before admission.

On admission, his body temperature was 40.0 °C, Glasgow Coma Scale score was 12 (E3-V4-M5), blood pressure was 166/77 mmHg, heart rate was 103 bpm, and oxygen saturation by pulse oximetry was 98% on room air. He was 174 cm tall and weighed 66 kg. Cerebrospinal fluid (CSF) analysis at the previous hospital showed a cell count of 4007/mm^3^, with 15% mononuclear cells and 85% polymorphonuclear cells, a protein level of 257 mg/dL, chloride of 123 mmol/L, and glucose of 37 mg/dL, findings highly suggestive of bacterial meningitis. Initial laboratory examination showed a white blood cell count of 19,190/μL and a C-reactive protein level of 14.9 mg/dL.

Magnetic resonance imaging (MRI) on admission demonstrated multiple enhancing lesions measuring less than approximately 1 cm scattered throughout the brain parenchyma (Figure 1). On repeat MRI obtained on day 6 of hospitalization, the lesions demonstrated more prominent enhancement, providing clearer visualization (Figure 2). Chest X-ray and computed tomography (CT) obtained on admission showed a mass-like lesion (approximately 5 cm) in the left lower lobe (Figure 3). Blood cultures obtained at the initiation of treatment later yielded *S. intermedius*. CSF cultures were negative. The multiplex meningitis/encephalitis panel detected human herpesvirus 6 (HHV-6); however, subsequent quantitative polymerase chain reaction testing for HHV-6 DNA was negative. CSF cytology was negative. Sputum culture could not be submitted because of difficulty in expectoration. The QuantiFERON-TB test and HIV-1/2 antibody confirmation test were negative, and serum assays for β-D-glucan, Aspergillus antigen, cryptococcal antigen and pneumococcal antigen were also negative. Oral examination revealed generalized moderate chronic periodontitis with abundant dental plaque accumulation and multiple periodontal pockets greater than 4 mm in depth. An ill-fitting maxillary denture with food impaction was also noted. These findings suggested poor oral hygiene and supported the oral cavity as the probable portal of bacterial entry. Transthoracic echocardiography revealed no findings suggestive of infective endocarditis. Because transthoracic echocardiography showed no findings suggestive of infective endocarditis and no clinical features raising a strong suspicion of endocarditis were present, transesophageal echocardiography was not performed. No other potential source of infection was identified in the cardiovascular or otorhinolaryngological systems. Based on the clinical presentation, bacteriological findings, and imaging results, the final diagnosis was multiple brain abscesses and a lung abscess.

Empirical antibiotic therapy was initiated with meropenem (2 g every 8 h) and vancomycin (1 g every 12 h) on the day of admission, considering the possibility of both brain and lung abscesses. On hospital day 7, the organism and its antimicrobial susceptibility were identified from the blood cultures obtained at admission. Subsequently, based on the susceptibility profile of the isolated organism, the antibiotic regimen was switched to ceftriaxone (2 g every 12 h). Fever of approximately 38 °C persisted until the seventh day after admission despite antibiotic therapy. Antimicrobial therapy was continued for a total of eight weeks. At the end of treatment, brain MRI and chest CT showed near-complete resolution of the abscesses. The patient became fully conscious by hospital day 30 and was able to walk independently at discharge. Follow-up MRI performed one month after completion of therapy showed no enhancing lesions in the brain.

## 3. Discussion

We report a case of multiple brain abscesses and a lung abscess that developed following COVID-19. *S. intermedius* is a Gram-positive coccus normally inhabiting the oral cavity, nasal passages, pharynx, and gastrointestinal tract. Unlike other oral streptococci, this bacterium has strong virulence and is known to form abscesses in organs such as the brain and liver [5]. In this patient, COVID-19 may have caused impairment of the immune barrier. Under this condition, *S. intermedius*, a pathogen associated with pre-existing periodontitis, could have entered the bloodstream from the oral cavity. The organism may then have spread hematogenously, resulting in multiple brain abscesses and a lung abscess.

Numerous reports have documented secondary infections following COVID-19. In a study of approximately 190,000 hospitalized COVID-19 patients, Murray and colleagues reported that 6.9% developed secondary infections; of these, 6.0% were bacterial, 0.9% viral, and 0.2% fungal [1]. Risk factors for secondary infection included older age (≥70 years), a history of chronic disease, lack of vaccination, invasive therapies, longer hospital stay, prior antibiotic treatment, and steroid use, among others [1,6,7].

The mechanism of secondary infection after COVID-19 is believed to involve multiple factors, including viral-induced immune dysfunction, tissue injury, and breakdown of mucosal barriers [8,9]. Severe acute respiratory syndrome coronavirus 2 (SARS-CoV-2) enters cells via the angiotensin-converting enzyme 2 (ACE2) [10]. ACE2 is not only an enzyme but also a functional receptor on cell surfaces. It is expressed in various tissues throughout the body, including intestinal epithelial cells, renal tubular cells, the gallbladder, cardiomyocytes, the eyes, vascular endothelial cells, and vascular smooth muscle cells [11]. SARS-CoV-2 triggers inflammation when it infects vascular endothelial cells via ACE2 [12]. In the cerebral vasculature, it has been demonstrated that the invasion of SARS-CoV-2 damages the integrity of tight junctions, the basement membrane, and adhesion molecules, leading to breakdown of the blood–brain barrier (BBB) [13]. Among central nervous system complications of COVID-19, a wide spectrum has been described, such as cerebral vascular disorders, encephalitis, encephalopathy, meningitis, and brain abscess [14,15]. There have been reports that, in COVID-19 cases presenting with neurological symptoms, disruption of the blood–cerebrospinal fluid barrier was observed in more than half of patients, even when cerebrospinal fluid samples were collected more than 30 days after the onset of neurological symptoms [16]. This observation raises the possibility that brain abscesses related to COVID-19 infection might develop after a delayed interval. In addition, delayed secondary bacterial infections and abscess formation have been reported following COVID-19, suggesting that infectious complications may occur beyond the acute phase of SARS-CoV-2 infection [17]. In the present case, brain abscesses developed approximately 20 days after the onset of COVID-19.

Similarly, in the oral cavity, high expression of ACE2 in the gingival sulcus has been reported, suggesting that mucosal barrier breakdown may predispose to secondary infection [18]. Taken together, the SARS-CoV-2-induced breakdown of immune barriers likely increased susceptibility to brain and lung abscess formation in this patient with a history of periodontitis. However, it is possible that the lung abscess had already begun to develop at the time of COVID-19 diagnosis. The patient experienced persistent fever and respiratory symptoms from the onset of COVID-19, and because chest imaging was not performed at that time, the presence of an early lung abscess could neither be confirmed nor excluded. Nevertheless, SARS-CoV-2-associated impairment of mucosal and immune barriers may have facilitated progression of the infection and subsequent hematogenous dissemination to the brain.

In addition, the combination of multiple ring-enhancing brain lesions and a pulmonary lesion may mimic metastatic malignancy, particularly lung cancer with brain metastases, potentially complicating the diagnostic process. Therefore, infectious etiologies should be carefully considered in the differential diagnosis when systemic inflammatory findings are present. In the present case, brain biopsy was not performed because the lesions consisted of numerous small microabscesses distributed throughout the brain parenchyma, making tissue sampling technically difficult and unlikely to provide substantial additional diagnostic benefit. Moreover, the diagnosis was supported by characteristic imaging findings, positive blood cultures for *S. intermedius*, and the presence of a concomitant lung abscess.

To better contextualize this case, we conducted a narrative literature review using PubMed/MEDLINE. Relevant articles were identified using the keywords “COVID-19”, “SARS-CoV-2”, “brain abscess”, “lung abscess”, and “*Streptococcus intermedius*”. Articles published in English were included, and the references of selected articles were also screened to identify additional relevant studies. Although several reports have described brain or lung abscesses following COVID-19, no cases involving the simultaneous occurrence of both have been reported. Among documented abscesses caused by *S. intermedius* after COVID-19, three cases involved the brain and one involved the lungs (Table 1) [3,19,20,21]. When comparing the present case with previously reported cases, notable differences include the younger age of the reported patients, a sinonasal portal of entry, and the presence of only one or two solitary abscesses in those cases. Solitary lesions likely reflected direct extension from adjacent structures such as the paranasal sinuses, whereas hematogenous spread, as suspected in our patient, tends to produce multiple lesions and can involve other organs. Multiple brain abscesses caused by *S. intermedius* are relatively rare, with only 11 cases reported (Table 2) [22,23,24,25,26,27,28,29,30,31,32]. Although some occurred in healthy young adults, many cases (including ours) had dental comorbidities such as periodontitis, caries, or recent dental treatment, which likely contributed to abscess formation. Another notable feature of our case is the presence of more than 80 tiny abscesses disseminated throughout the brain parenchyma, representing a state of multiple brain microabscesses. Such numerous lesions are exceedingly rare; we identified only two comparable cases in the literature [31,32]. Notably, the report by Virtanen et al. did not provide information on abscess size or brain imaging, preventing definitive assessment [29]. Therefore, the present case represents an exceptionally rare manifestation of *S. intermedius* infection.

Brain abscess formation is classically described in four stages, i.e., early cerebritis, late cerebritis, early capsule formation, and late (mature) capsule formation, during which neutrophils play an essential role in organizing purulent material and forming a capsule [33]. When neutrophils are depleted or functionally impaired, this “consolidation” of pus fails to occur, preventing the development of a typical encapsulated abscess. Consequently, multiple brain microabscesses are more commonly observed in conditions such as neutropenia, neutrophil dysfunction, immunocompromise, or fungal infections [34,35]. Although our patient did not have an immunocompromised background, previous studies have reported impaired leukocyte chemotaxis following SARS-CoV-2 infection [36]. In the present case, COVID-19-related vascular and immunological disturbances in the central nervous system may have facilitated hematogenous dissemination of *S. intermedius*, potentially contributing to the formation of multiple brain microabscesses.

As the patient had periodontitis, which can directly cause such abscesses, we cannot completely rule out the possibility that the development of brain and lung abscesses was unrelated to the prior COVID-19 infection. Although a causal relationship cannot be established, the temporal association and the reported effects of SARS-CoV-2 on immune and mucosal barriers raise the possibility that COVID-19 contributed to the progression and dissemination of infection in this patient. Further accumulation of similar cases of secondary infection after COVID-19 is warranted.

## 4. Conclusions

We report a patient who developed multiple brain abscesses and a lung abscess after COVID-19. Although the relationship between COVID-19 and abscess formation remains uncertain, clinicians should consider the possibility of severe secondary bacterial infection when fever or respiratory symptoms persist beyond the typical course of COVID-19, particularly in patients with pre-existing oral disease such as periodontitis.

## Figures and Tables

**Figure 1:**
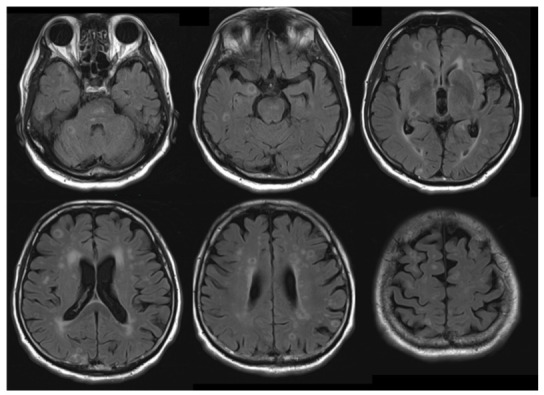
Axial fluid-attenuated inversion recovery (FLAIR) MRI of the brain on admission. Axial FLAIR images demonstrate numerous small ring-like lesions (<1 cm) distributed throughout both cerebellar hemispheres and both cerebral hemispheres.

**Figure 2:**
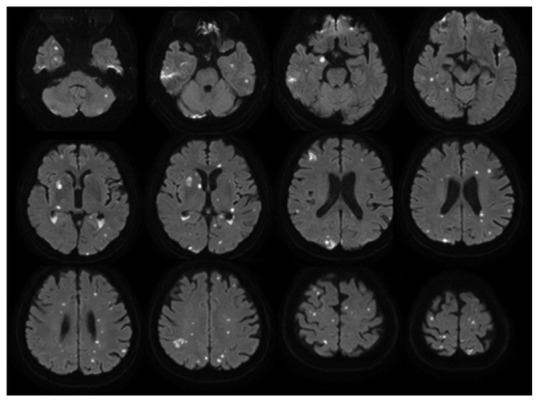
Axial brain diffusion-weighted imaging (DWI) MRI on hospital Day 6. DWI demonstrates numerous microlesions distributed throughout both cerebellar hemispheres and both cerebral hemispheres.

**Figure 3:**
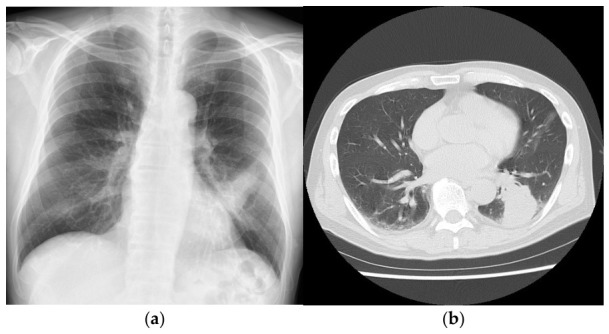
Initial chest X-ray and computed tomography (CT) on admission. (**a**) Chest X-ray shows a mass-like lesion in the left lower lobe; (**b**) Chest CT with lung windows shows a mass-like lesion in the left lower lobe.

**Table 1:** Comparison of reported cases of *Streptococcus intermedius* brain or lung abscesses following COVID-19.

Case	Age (y)	Sex	Medical Background	Abscess Location	Number of Abscesses	Presumed Portal of Entry	Specimens Yielding Pathogens	Time from COVID-19 Diagnosis to Abscess Onset
Kunapaisal et al. [3]	13	M	None	Right frontal lobe	1	Paranasal sinusitis	Pus	0 days
Griffin et al. [19]	15	M	None	Right frontal lobe (epidural and subdural)	2	Paranasal sinusitis	Pus	7 days
Blitz et al. [20]	Adolescent ^a^	M	None	Left frontal lobe (subdural), right frontal lobe	2	Paranasal sinusitis	Pus, blood	Not reported
Bueno et al. [21]	25	F	Pregnant	Right lower lobe	1	Airway	Pus	N/A
Present case	75	M	Cholelithiasis	Whole brain, left lower lobe	≥80 (brain), 1 (lung)	Periodontitis	Blood	17 days

Abbreviations: M, male; F, female; N/A, not available. ^a^ Age 10–19 years; exact age not reported. Data extracted from previously published reports [3,19,20,21].

**Table 2:** Comparison of reported cases of multiple brain abscesses caused by *Streptococcus intermedius*.

Case	Age (y)	Sex	Medical Background	Abscess Location and Number	Presumed Portal of Entry	Specimens Yielding Pathogens
Maliyil et al. [22]	21	M	None	Brain: 3; spleen: 1	Unknown	Blood, spleen pus
Reyes et al. [23]	47	M	Diabetes, dental caries	Brain: ≥3; lung: 1; liver: 1	Dental caries (aspiration)	Lung and liver pus
Isern et al. [24]	43	M	Unknown	Brain: ≥4	Streptococcal pharyngitis (suspected)	Blood, brain pus
Bhatia et al. [25]	26	M	None	Brain: ≥8; spinal cord: ≥1	Pneumonia	CSF, LN
Fransson et al. [26]	34	M	None	Brain: ≥5	Periodontitis	Brain pus
Herskovitz et al. [27]	22	M	None	Brain: 13	Tongue piercing/Oral piercing site infection	Brain pus
Swied et al. [28]	32	M	Hypertension, recent pneumonia	Brain: ≥3	Esophageal perforation, dental caries	Brain pus
Virtanen et al. [29]	18	M	Histoplasmosis-induced pericarditis (7 years before)	Brain: ≥30; spinal cord: 1	Unknown	Spinal cord pus
Sayyahmelli et al. [30]	25	F	Partial OTCD	Brain: 2	Tooth extraction/oral source	Brain pus
Ohara et al. [31]	50	M	None	Brain: ≥30	Pneumoniae, paranasal sinusitis	CSF
Yao et al. [32]	67	M	None	Brain: ≥40; lung: 1	Pneumonia	CSF, BALF
Present case	75	M	Cholelithiasis, COVID-19	Brain: ≥80; lung: 1	Periodontitis	Blood

Abbreviations: BALF, broncho-alveolar lavage fluid; CSF, cerebrospinal fluid; LN, lymph node; OTCD, ornithine transcarbamylase deficiency. For cases in which the exact number of abscesses was not provided in the original report, the minimum number of lesions (≥) was estimated based on the description of affected brain regions and/or review of published MRI/CT images. Data were extracted from previously published reports [22,23,24,25,26,27,28,29,30,31,32].

## Data Availability

No new data were created or generated in this study. The case report is based on the patient’s clinical course and existing medical records, and the literature review is based on previously published studies. Therefore, data sharing is not applicable to this article.

## Abbreviations

The following abbreviations are used in this manuscript:

ACE2	Angiotensin-converting enzyme 2
BBB	Blood–brain barrier
COVID-19	Coronavirus disease 2019
CSF	Cerebrospinal fluid
CT	Computed tomography
HHV-6	Human herpesvirus 6
MRI	Magnetic resonance imaging
SARS-CoV-2	Severe acute respiratory syndrome coronavirus 2
